# Up-regulation of miR-370-3p restores glioblastoma multiforme sensitivity to temozolomide by influencing MGMT expression

**DOI:** 10.1038/srep32972

**Published:** 2016-09-06

**Authors:** Yong-tao Gao, Xiao-bing Chen, Hong-lin Liu

**Affiliations:** 1 Department of Neurosurgery, Huaihe Hospital of Henan University, 475000, Kaifeng, Henan Province, 475000, China

## Abstract

MicroRNAs (miRNA) are believed to play an important role in glioblastoma multiforme (GBM)chemotherapy. Our study aims to investigate potential miRNA biomarkers in GBM. Sixty GBM patients, which were given temozolomide (TMZ) chemotherapy and recurrent radiotherapy, were recruited. miRNA array was performed in cancerous and in paired normal tissues. Microarray results were further validated by a quantitative real-time PCR in selected tissues and GBM cell lines. TMZ resistance cells were developed and cell proliferation along with colony formation assays was determined. Our study employed H2AX formation and flow cytometry to analyse the role of miRNA in DNA damage and apoptosis. Our study illustrated 16 miRNA in which 9 were up-regulated and 7 down-regulated. and their differential expression were demonstrated in a recurrent GBM tissue. Among them, miRNA-370-3p demonstrated the highest level of down- regulation in tissues and in TMZ resistance cells. miRNA-370-3p mimic increased its expression and sensitivity of GBM cells to TMZ by suppressing the self-reparative ability of tumour cell DNA. O^6^-methylguanine-DNA methyltransferase (MGMT) was identified as the direct target gene of miR-370-3p, and it was found to be inversely correlated with miR-370-3p expression in tissue samples obtained. Thus, our study demonstrated a critical clinical role of an up-regulated miR-370-3p expression in glioblastoma multiforme chemotherapy sensitivity.

Approximately 70% of human malignant primary brain tumours are gliomas^1^. Unfortunately, the highest form of malignant glioma is the glioblastoma mulitforme (GBM), which has the highest prevalence among humans. The treatment protocol routinely selected by physicians is surgical resection combined with the use of temozolomide and radiation therapy, which has shown its potential to increase the survival rate of GBM patients^2^. However, the disease prognosis remains dismal due to acquired chemotherapy resistance resulting in a recurrent tumour growth^2^. The diffuse infiltrative nature of GBM that stimulates cells to deeply invade the dense network of brain structure is associated with tumour recurrence and resistance^3^. Despite the advancements in understanding of glioma biology, the molecular mechanisms underlying resistance have yet to be fully explored. Since acquired TMZ chemo-resistance occurs in more than 90% of recurrent high-grade gliomas^4^, investigating the underlying mechanisms involved in chemo-resistance carry the potential to improve the survival rate of patients suffering from GBM^5^.

MicroRNA (miRNA) are short, single-stranded non-coding RNA (consisting of 22 nucleotides) that widely participate in many post-transcriptional regulation processes and are quintessential in regulating various cellular processes^6^. Recently, there has been an emerging interest in miRNA and their involvement in tumorigenesis and progression of various cancers, including GBM^7^. Previous reports have shown that certain miRNA are implicated in an acquired TMZ resistance^8^. For example, a study has reported that TMZ resistance in human glioma cell lines occur due to an up-regulation of miR-195, miR-455-3p and miR-10a^9^. In another human tissue study, a group of GBM patients were treated with a TMZ which exhibited a down-regulation of miR-181b and miR-181c^10^. Our study aims to provide both an investigative and a potential role of miRNA in the management of recurrent GBM. Furthermore, our study seeks to provide evidence that miRNA carries a potential therapeutic benefit towards chemo-resistance.

## Material and Methods

### Patients and tissue samples

GBM tissue samples (31 male, 29 female, average age 51) were collected from July 2010 to July 2014 from the Huaihe Hospital. All patients’ samples underwent the TMZ chemotherapy following radiotherapy and recurrent treatment prior to surgery (Table 1). Fresh, frozen, human non-neoplastic brain tissues (13 individuals) were obtained from the Department of Pathology at the Huaihe Hospital. Three neuropathologists performed histo-pathological diagnoses on all the tissue samples obtained.

### Ethics statement

The study protocol was approved by the Ethics Committee of Henan University. Informed consent was obtained from all patients prior to surgery. Specimens and all experimental procedures were handled and carried out in accordance with the approved guidelines.

### Cell lines

Primary normal human astrocytes (NHA) was obtained from the Cell Bank of the Chinese Academy of Sciences (Wuhan, People’s Republic of China). Seven common human glioma cell lines U87, LN229, LN18, SHG44, U373 and U251, T98G were obtained from the American Type Culture Collection (ATCC, Manassas, VA, USA). Cells were incubated in a humidified incubator with 5% CO_2_ at 37 °C supplied with DMEM culture medium (Gibco, Carlsbad, CA, USA) containing 10% fetal bovine serum (Gibco, USA). All cell lines were tested for mycoplasma contamination before use.

### Determination of differentially expressed microRNAs

The TaqMan array human microRNA card set v3.0 (Applied Biosystems, Calsbad, CA, USA)was performed to compare miRNA expression levels between tumor tissues and control. The set provided an accurate quantitative value of 754 human microRNAs and aligned with the Sanger miRBase v20. cDNA samples were obtained from different groups, which were loaded on cards and run on real-time system. Data was exported and analyzed in the SDS v2.0 software. (Applied Biosystems, USA) as previous description^10^. Briefly, the lower signal genes (<300 pixels) were excluded from the results. Statistical difference was compared by using the two sided t-test with unequal variance assumptions. The false discovery rate was performed to correct for multiple hypothesis testing. At least three independent samples were selected to calculate the average density of hybridization signals. Differentially expressed genes were selected using both a false discovery rate of less than 0.01 and a fold-change greater than 2.0 or less than 2.0.

### TMZ cytotoxicity assay

1 × 10^3^ cells/per well were seeded on 96 well plates in triplicates for 48 hours to achieve 70% confluence, followed by the TMZ (Sigma Aldrich, St. Louis, MO, USA) treatment at various concentrations (0 μM, 5 μM, 10 μM, 15 μM, 20 μM, 25 μM, 30 μM). Commercial cell proliferation assay kit (Promega, Madison, WI, USA) was utilized to evaluate the number of viable cells in the TMZ cytotoxicity assay.

### Real-time PCR

Purified RNA and miRNA was extracted using the miRNeasy kit (Qiagen, Hilden, Germany) following the manufacturer’s protocol; the specimen was stored at a temperature of −80 °C. The quality and quantity of samples were determined with the Nanodrop (Thermolife science, Hercules, CA, USA). The qRT-PCR was performed on ABI 7900 fast sequence detection system (Applied Biosystem, Calsbad, CA, USA) with a two-step SYBR green fluorescent transcription kit (Takara, Dalian, China), and microRNA Locked Nucleic Acid PCR primers specific for miR-370-3p were utilized (Exiqon, Denmark). Our study used the following PCR time related conditions:: 95 °C for 10 minutes, 40 cycles of 95 °C for 15 seconds, 60 °C for 1 minute and finally an elongation step at 72 °C for 30 seconds. RNU48 was chosen as an internal control to calculate the relative expression in tumor tissues and healthy control tissues. Each RNA sample was analyzed three times with the following primers:

Human MGMT primers: forward 5′-GAGGCGTGGCAGACTATGC-3′; reverse 5′-CTTGTACTCCGTCAGCGTGA-3′.

Human miR-370-3p primers: forward 5′-TGTAACCAGAGAGCGGGATGT-3′; reverse 5′-TTTTGGCATAACTAAGGCCGAA-3′.

### Measurement of apoptosis by flow cytometry

Single cell suspensions were collected from different treatment groups, and the percentage of cells in apoptosis were observed with the Annexin/PI staining kit following the manufacturer’s instructions (Abcam, Cambridge, UK). The sample was kept on ice for further flow cytometric analysis. All cell types undergoing apoptosis were quantified by staining with the Annexin V-FITC and PI. By conjugating FITC to Annexin V, it was possible to identify and quantify apoptotic cells on a single-cell basis by flow cytometry. For this experiment, the CellQuest software was used to quantify and analyze cells with early apoptotic (FITC^+^PI^−^) and late apoptotic or necrotic cells (FITC^+^PI^+^).

### Transient transfection of miR-370-3p mimics

GenePharma (Shanghai GenePharma Co. Ltd., Shanghai, China) was used as the source for miR-370-3p mimics and negative miRNA controls. The Lipofectamine 2000 (Invitrogen, Calsbad, CA, USA) was used to perform transfections. Cells were transfected with miR370 controls and mimics were employed into a final 50 nM solution for 48 h.

### Luciferase assay

The direct binding effect of miR-370-3p and its predicted target MGMT was validated with the dual luciferase reporter assay (Abcam, Cambridge, UK). Test cells were transfected with the miR-370-3p mimic or negative control by using the Lipofectamine 2000 (Invitrogen, USA) as described previously^11^.

### Western blot analysis

Proteins were extracted using a commercial extraction buffer (containing protease inhibitor(Cell Signaling Technology, Beverly, MA, USA). Proteins were separated by 10% SDS-PAGE and transferred to a PVDF membrane under semi-dry conditions following 4 hour blocking with 5% skim milk in TBST at room temperature. Membranes were incubated with a mouse monoclonal primary antibodies (MGMT and H2AX, Cell signaling, USA) overnight at 4 °C and then were washed and incubated with secondary antibodies for 1 hour at room temperature and analyzed with an enhanced chemiluminescence substrate (BioRad, Hercules, CA,USA).

### Colony formation assay

Colony formation assay was conducted to validate the influence of miR-370-3p on cell proliferation with the TMZ treatment. 1 × 10^5^ cells/per well were seeded onto 24-well plates and transfected with a vector which included miR-370-3p or a mock control. After 48 h incubation, 500–1000 cells were counted and seeded in six-well plates until formation of visible colonies was observed. Colonies were fixed and stained; those colonies containing more than 50 cells were counted. The numbers of cloneswere examined using macroscopic observation.

### γ-H2AX foci formation assay

1 × 10^5^ cells were cultured in a flask to achieve 70% confluence. The flask was fixed with 2% paraformaldehyde followed by 0.5% Triton X-100 permeability (Sigma, MO, USA). Suspended cells were incubated with a mouse monoclonal primary antibody target to Ser 139 phosphorylation of H2AX (Cell Signaling Technology, USA, 1:400) at a room temperature for 4 hours and were incubated with Alexa Fluor 488-labelled secondary antibody (Cell signaling1:1000). Cells were finally placed on the microscope slide with a cover slip containing a mounting medium, and samples were analyzed using a fluorescence microscope (Zeiss, Germany).

### Database

The Catalogue Of Somatic Mutations in Cancer database at the Sanger Institute (COSMIC, http://www.sanger.ac.uk/genetics/CGP/cosmic/), the Cancer Genome Atlas database (TCGA, http://cancergenome.nih.gov), and the Chinese Glioma Genome Atlas (CGGA) databases were used to collect miRNA and methylation information in human malignant glioma.

### Statistical analysis

SPSS 13.0 (SPSS Inc., Chicago, IL, USA) was used for all data analyses. Our study made use of the Student’s t-test to determine the difference between the study and control samples. The Pearson correlation analysis was performed to determine an association for the study. All numerical data was expressed as Mean ± SEMs. Each experiment was repeated at least twice or in triplicates. A *P* value of less than 0.05 was considered statistically significant.

## Results

### Demographics of study population

All patients experienced surgical treatment with a total resection or a partial resection for recurrent tumors. As shown in Table 1, a variety of clinical evaluations were recorded by a certified health care provider to assess every patient’s clinical features. Most tumors (51%) were located in the right hemisphere of the brain to conduct analysis for our study.

### Comparison of miRNA expression profiling in recurrent GBM and unpaired non-cancerous tissues

**Figure 1:**
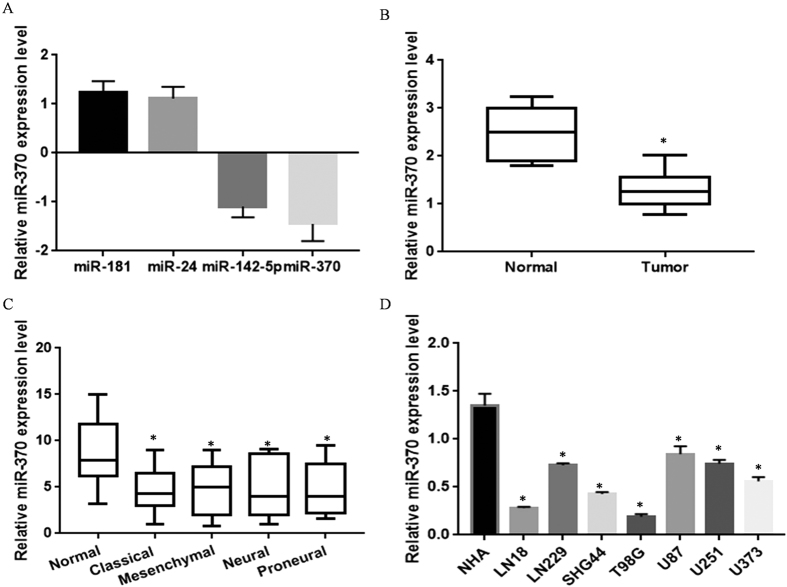
Differential miRNA expression profiles in GBM tissues and cells. (**A**) Four randomly selected and differentially expressed miRNA from an array data were confirmed by the qRT-PCR in triplicate. (**B**) Summarized miR-370-3p expression in all samples. MiR-370-3p expression level was lower in GBM tissue (compared to paired non-cancerous tissue). RNU48 served as an internal control. *P < 0.05 compared with parental cells. (**C**) MiR-370-3p expression data in four molecular subtypes (Proneural, Neural, Classical and Mesenchymal) of GBM samples from The Cancer Genome Atlas (TCGA) portal. *P < 0.05 compared with normal cells. (**D**) miR-370-3p level in seven glioblastoma multiforme (GBM) cell lines and primary normal human astrocytes (NHA). *P < 0.05 compared with normal NHA.

Microarray results revealed a total of 16 differentially expressed miRNA (tissue/normal > 1.5) in primary GBM (Table 2). The four most highly up regulated and down regulated miRNA were validated by the RT-PCR (Fig. 1A). miR-370-3p exhibited the greatest change in the array and in the RT-PCR results and expression of miR-370-3p was detected in 60 recurrent GBM samples and in unpaired normal tissues; the expression of miR-370-3p was normalized to internal β-actin controls. Results showed that miR-370-3p expression levels were significantly lower in recurrent GBM tissues than non-cancerous tissues (Fig.1B). Further data analysis from TCGA revealed that miR-370-3p was expressed at low level across in all four molecular subtypes (Proneural, Neural, Classical and Mesenchymal comparable to normal brain cells (Fig. 1C).

### Up-regulation of miRNA-370 suppresses GBM cell viability *in vitro*

**Figure 2:**
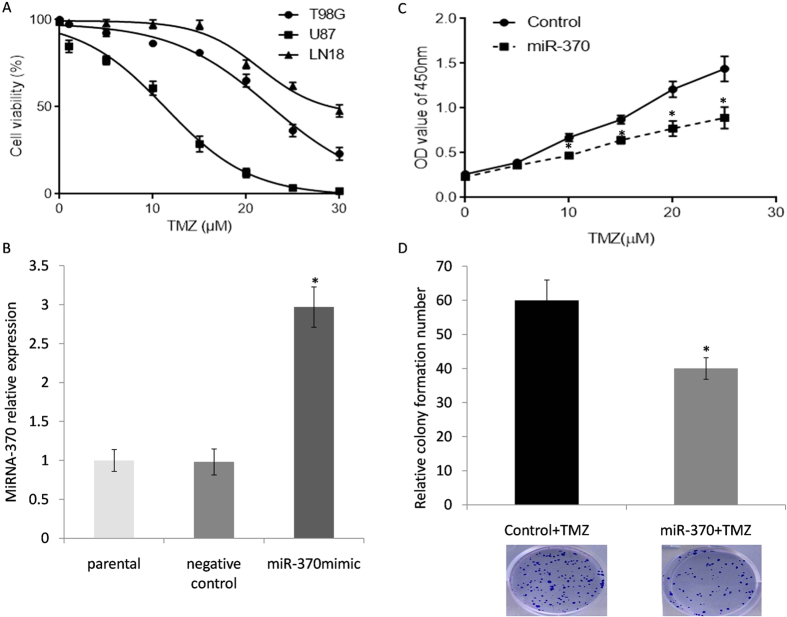
MiR-370-3p sensitized the response of glioma cells to temozolomide. (**A**) Innate sensitivity to TMZ in two lowest (LN18, T98G) and one highest miR-370-3p expressed cell line (U87). (**B**) LN18 was transfected with miR-370-3p mimics and scrambled control miRNA. (**C**) Cell proliferation confirmed that miR-370-3p mimic cells were more sensitive to TMZ than controls (*P < 0.05). (**D**) Cell colony formation miR-370-3p mimic cells were more sensitive to TMZ than controls (*P < 0.05). The colony forming ability of miR-370-3p mimics cells were significantly decreased following 24 hours of the TMZ treatment.

*In vitro*,miR-370-3pwas declined in all GBM cell lines that were tested in comparison with normal controls. Among them, LN18 cells demonstrated the lowest miR-370-3p expression level and were chosen for the next functional study (Fig. 1D, *P* < 0.05). Also, we compared the cell sensitivity to TMZ in two lower (LN18, T98G) and one higher miR-370-3p expressed cell line (U87). LN18 and T98G which had lowest miR-370-3p levels showed relative resistance (IC_50_ = 31.26 μM) to TMZ compared to U87 (IC_50 _= 10.97 μM) (Fig. 2A). The expression of miR-370-3p was validated by the qRT-PCR. It was found that the level of miR-370-3p had increased significantly in cell lines treated with the mimic (Fig. 2B). The MTT and colony formation assays were used to evaluate the impact of miR-370-3p on cell proliferation after treatment with multiple doses of TMZ. A statistically significant decrease in proliferation and colony formation percentage was observed after an up-regulation of miR-370-3p (Fig. 2C,D). The data in these figures suggests that miR-370-3pcarries the potential to inhibit cell proliferation *in vitro*.

### Up-regulation of miRNA-370 promoted TMZ induced DNA damage and apoptosis

**Figure 3:**
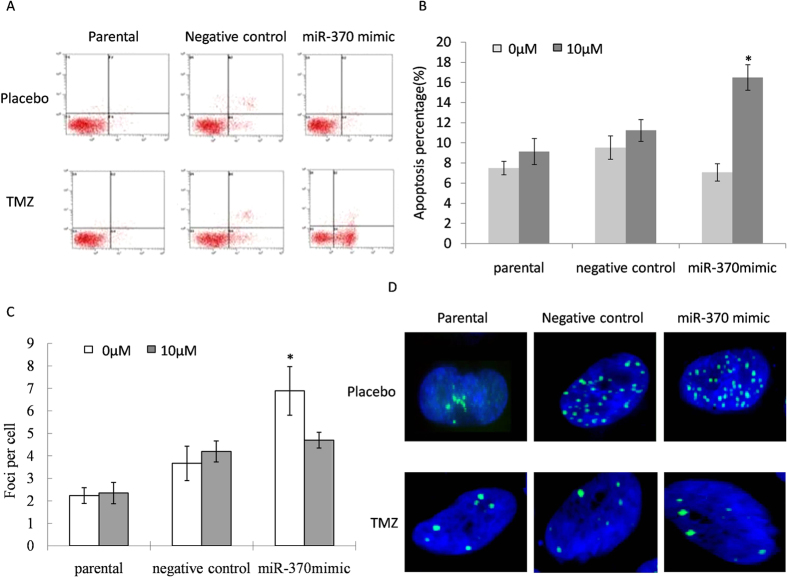
Up-regulation of miRNA-370-3p promoted TMZ induced DNA damageand apoptosis. (**A,B**) Apoptosis assays demonstrated that an up regulation of miR-370-3p increased TMZ-induced cell death in the LN18 cells (*P < 0.05). (**C**) The mean number of foci per cell for various doses is shown. Number differences of foci in groups were labelled with asterisks. Error bars represent SEMs. (**D**) γ-H2AX foci (green) in non-chemotherapy treatment (a–d) and miR-370-3p treatment cells (e–h) followed different TMZ doses (0 μM, 1 μM, 5 μM, 10 μM); nuclei were stained with 4,6-diamidino-2-phenylindole (blue). Data represents the mean of three independent experiments; bars: SEMs.

The data suggested that miR-370-3p suppresses TMZ-induced DNA repair signal of GBM cells. Afterwards, we investigated the effect of miR-370-3p on the apoptosis of glioma cancer cells after the TMZ treatment (Fig. 3A). Results showed that miR-370-3p statistically increased the rate of apoptosis (Fig. 3B).The number of histone γ-H2AX foci was used to investigate the effect of miR-370-3p on cells that carry DNA double breaks from randomly selected 500 nuclei in different treated groups (Fig. 3C). The formation of γ-H2AX was significantly down-regulated after treatment with TMZ in miR-370-3p mimic transfected cells (Fig. 3D).

### Direct binding gene of miR-370-3p and its association with target gene expression levels

**Figure 4:**
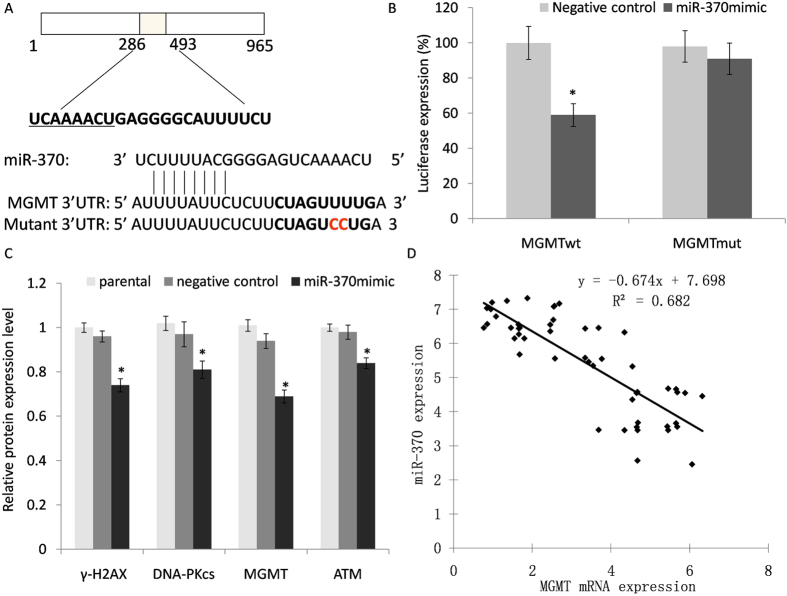
MiR-370-3p binds to the 3′-UTR of MGMT mRNA and influences DNA pathway protein expression. (**A**) A luciferase report system vector was constructed, which included a wild-type 3′-UTR fragment of MGMT or mutant miR-370-3p binding sequence. (**B**) Luciferase activity was detected in LN18 cells co-transfected with a luciferase reporter system and miR-370-3p or miR-NC. (**C**) Following TMZ treatment, DNA pathway proteins in different treatment of GBM cells were evaluated with the Western blot analysis. β-actin was used as an internal control. (**D**) Correlation of miR-370-3p and MGMT expression in patients. Each experiment was performed in triplicate with *P < 0.05, compared to negative controls.

To further elucidate the mechanism by which miR-370-3p affected cellular function, we screened for potential targets of miR-370-3p using four target prediction programs with different algorithms: DIANA-MicroT, TargetScan, Miranda and PicTar. All potential targets predicted by more than one of these programs were identified. We selected MGMT for further study because of its recognized role in GBM tumor biology. A luciferase reporter vector containing MGMT 3′ UTR with miR-370-3p binding sites was generated to analyze whether miR-370-3p binds to the 3′ UTR of the target miRNA (Fig. 4A). A mutant reporter vector was also generated, which contained MGMT3′ UTR with a mutation at the putative binding site for miR-370-3p. As shown in Fig. 4B, luciferase activity in miR-370-3p mimic transfected cells significantly decreased compared to the control cells (*P* < 0.05). Simultaneously, no observed luciferase alteration was observed in cells that were transfected with the mutant 3′ UTR constructs. Our results demonstrated that the expression of DNA-Pkcs, γ-H2AXand MGMT were suppressed in miR-370-3p transfected cells compared with non-transfected cells after the TMZ treatment (Fig. 4C). In contrast, the expression levels of ATM failed to demonstrate any significant changes. Therefore, the results suggested that restoration of TMZ sensitivity in cells is associated with a reduction in DNA repair capacity following miR-370-3p treatment. In order to further analyse the correlation between miR-370-3p and MGMT expression in glioma cancer tissue, our study examined miR-370-3p and mRNA of MGMT in all glioma and their correspondingly matched normal adjacent tissues. The Pearson’s correlation analysis showed a significant negative correlation between the presence of MGMT mRNA and miR-370-3p (r = −0.826, *P* < 0.05) (Fig. 4C).

## Discussion

TMZ is currently the most common oral chemotherapy drug to treat GBM patients^2^. However, over the past few years, variable individual sensitivity to TMZ has been frequently reported in GBM patients^11^,^12^,^13^. It is important to understand how tumors respond to TMZ. Moreover,, researchers can determine the genetic markers. which can identify patients that are most and least likely to benefit from the TMZ treatment. MiRNA has been shown to play a crucial role in the post-transcriptional regulation of genes involved in drug response processes^14^,^15^. In our clinical data, following the TMZ treatment, recurrent GBM patients demonstrated significant miRNA alterations. Among them, miR-370-3p showed the highest decrease. Moreover, the down regulation of miR-370-3p exists globally in several GBM cell lines. Our data revealed a potentially important role of miR-370-3p in the GBM treatment chemotherapy. Also, previous studies provided a strong evidence that miR-370-3p is involved in tumorigenicity in various types of cancers^16^,^17^. For example, an over expression of miR-370-3pwas found to be associated with G1/S cell arrest in prostate cancer cells^18^. Furthermore, an up regulation of miR-370-3phas been correlated with an advanced gastric carcinoma metastasis via regulating the transcription level of the transforming growth factor-β receptor II (TGFβ-RII) gene^19^. Therefore, our study findings add to the existing literature by illustrating an additional key role for miR-370-3p in brain tumours.

Some reports have shown that certain miRNA are implicated in an acquired TMZ resistance^20^,^21^. Previous research has shown that miR-195, miR-455-3p and miR-10a were up regulated in a set of TMZ resistant laboratory-generated human glioma cell lines^9^. In contrast to these findings, another study found that GBM patients, who had responded to concomitant radiotherapy with TMZ, exhibited a down regulation of miR-181b and miR-181c^10^. *In vitro* functional results, from our study, indicated that an up- regulation of miR-370-3p sensitizes GBM cells to TMZ, which causes suppression in the ability of DNA repair, activation of H2AX, enhanced rate of apoptosis, and suppressed growth of glioma cells. Our study is among the first to demonstrate the effects of an up-regulated miR-370-3pin the TMZ-resistant GBM. Our findings suggest that an up regulation of miR-370-3p could be used as a chemotherapy adjunct (for example, for patients who have lower miR-370-3p levels serologically or within their tumours). Other miRNA candidates associated with TMZ resistance in GBM include miR-195, miR-455-3p, miR-10a, miR-181b and miR-181c^22^,^23^,^24^. These may be further explored for similar clinical applications alone or in combination with miR-370-3p.

Regarding the mechanism of TMZ, upon its entrance into the central nervous system, it immediately converts into an active metabolite, 3-methyl-(triazen-1-yl)imidazole-4-carboxamide (MTIC). MTIC then reacts with water, and liberates 5-aminoimidazole-4- carboxamide as well as the highly reactive methyldiazonium. The new and active methyl diazonium cation methylates DNA at N7 position (N7-MeG) of areas rich in Guanine, as well as the N7 positions of N3 Adenine (N3-MeA) and O6 Guanine (O6-MeG) residues^25^. The DNA repair enzyme, O6-methyl-guanine-methyltransferase (MGMT), then acts to remove any resulting methyl adducts^26^,^27^. According to the Cosmic record, the panel of GBM cell lines showed a heterogeneous MGMT methylation status which resulted in a different response towards the TMZ treatment. e.g. LN18 and T98G showed unmethylation of MGMT, whereas, the other two cell lines, U87 and U251, had methylated promoter region status for MGMT. Afterwards, the process expresses an innate high level of MGMT expression, which allows for the existence of a resistant phenotype by inhibiting TMZ-related effects.

Hegi *et al*. suggested that epigenetic silencing of the MGMT gene through promoter methylation was associated with a longer survival in GBM patients who had received TMZ or other DNA alkylating treatments^28^. Currently, MGMT is considered to be a strong characterised modulator of chemo-resistance in GBM^29^,^30^,^31^. The primary damage caused by TMZ is the creation of O6-MeG DNA adducts. If these adducts are left unrepaired by MGMT, the adducts will produce incorrect pairings of bases and trigger intervention of a mismatched repair system^32^.

Our data confirmed the direct binding and regulatory effects of miR-370-3p on MGMT. Our study demonstrated that MGMT expression was significantly associated with miR-370-3p expression in patients’ samples. Together with other DNA repair systems, miR-370-3p could work as a chemotherapy adjunct to improve the TMZ therapy.

In conclusion, our study has shown that miR-370-3p is significantly down-regulated in a recurrent glioma cancer tissue. We have also shown that an over-expression of miR-370-3p could effectively confer TMA induced cell proliferation, DNA repair inhibition and could enhance apoptosis of glioma cancer cells *in vitro*. MiR-370-3p could negatively regulate the expression of the MGMT gene. Thus, our results highlight a potential therapeutic role of miR-370-3p in the treatment of brain cancers.

## Additional Information

**How to cite this article**: Gao, Y.- *et al*. Up-regulation of miR-370-3p restores glioblastoma multiforme sensitivity to temozolomide by influencing MGMT expression. *Sci. Rep*. **6**, 32972; doi: 10.1038/srep32972 (2016).

## Figures and Tables

**Table 1:** Demographics of study patients.

Clinical parameters	No. of case (%)
Gender	
Male	31 (51.0)
Female	29 (49.0)
Median age at diagnosis Age	51 (21–72) years
Median KPS	78(30–100)
Tumour localization	
Right hemisphere	29 (51.0)
Left hemisphere	27 (47.1)
Both hemispheres	4 (1.9)
Frontal	21 (41.1)
Temporal	18 (31.4)
Parietal	12 (25.5)
Occipital	9 (17.6)

Note: Karnofsky Performance Score (KPS).

**Table 2:** MiRNA differentially expressed in GBM tumour tissue compared to normal tissues.

No.	MiRNA name	Mean fold change	P-value
1	miR-181b	3.11	0.0296
2	miR-24	3.04	0.0135
3	miR-181	2.86	0.0024
4	miR-146a	2.61	0.0169
5	miR-126	2.44	0.0036
6	miR-424	2.21	0.0095
7	miR-155	1.96	0.0133
8	miR-505	1.84	0.0394
9	Let-7e	1.62	0.0295
10	miR-370-3p	−3.41	0.0069
11	miR-142-5p	−3.02	0.0034
12	miR-34c	−2.73	0.0016
13	miR-77	−2.46	0.0108
14	miR-130a	−2.19	0.0072
15	miR-22	−1.63	0.0183
16	miR-299-5p	−1.59	0.0236

Note: Positive and negative fold change scores means significant down regulation and up regulation, respectively, in tumors.
